# Acute ischemic stroke during TAVI promptly managed with mechanical thrombectomy: the importance of peripheral interventional skills and multidisciplinary collaboration

**DOI:** 10.1016/j.jccase.2026.03.012

**Published:** 2026-04-15

**Authors:** Marcello Marchetta, Giulio Russo, Gianluca Massaro, Maria Crapanzano, Daniela Benedetto, Giuseppe Massimo Sangiorgi

**Affiliations:** a Department of Biomedicine and Prevention, Cardiology Unit, Policlinico Tor Vergata, University of Rome, Rome, Italy

**Keywords:** Case report, Transcatheter aortic valve implantation, Ischemic stroke, Procedural complication, Mechanical thrombectomy, Aortic stenosis

## Abstract

Transcatheter aortic valve implantation (TAVI) carries a small but serious risk of periprocedural stroke. Prompt recognition and rapid management are essential to prevent permanent neurological injury. We describe an acute ischemic stroke occurring during TAVI for bicuspid aortic stenosis, immediately identified by the interventional cardiologist through carotid angiography. The patient developed sudden left hemibody weakness and aphasia after balloon predilatation, and angiography revealed an occlusion of the right middle cerebral artery (M1). While maintaining hemodynamic stability with valve implantation, the operator restored partial flow in the ophthalmic artery and activated the stroke team. Mechanical thrombectomy was rapidly performed using an aspiration catheter, achieving complete recanalization, with a modified Thrombolysis in Cerebral Infarction grade 3 within 30 min of symptom onset. Post-procedural imaging confirmed the absence of hemorrhage, and neurological deficits improved markedly. This case highlights how immediate in-laboratory carotid angiography performed by the structural interventional cardiologist can significantly shorten the diagnostic-to-reperfusion interval and facilitate ultra-rapid multidisciplinary rescue in periprocedural stroke.

**Learning objective:**

Immediate in-laboratory carotid angiography by the structural operator may significantly shorten time-to-reperfusion in periprocedural stroke. Peripheral interventional skills are essential for structural cardiologists, enabling them to promptly recognize and address extracardiac complications. Multidisciplinary collaboration between interventional cardiologists, neurologists, and interventional radiologists is vital to achieve optimal outcomes in life-threatening complications. The absence of cerebral embolic protection did not compromise the outcome, consistent with recent randomized evidence showing no significant reduction in clinical stroke with routine use of protection devices.

## Introduction

Stroke is one of the most feared complications of transcatheter aortic valve implantation (TAVI), with an incidence consistently reported around 2–4% at 30 days [1], [2]. Beyond acute morbidity and mortality, periprocedural stroke has a significant impact on long-term functional outcomes, quality of life, and healthcare expenditures [3]. The 2025 European Society of Cardiology (ESC)/European Association for Cardio-Thoracic Surgery guidelines recommend that TAVI be performed in specialized centers with immediate access to multidisciplinary expertise and neurovascular rescue strategies, reflecting the devastating consequences of cerebrovascular complications. Mechanical thrombectomy is the established treatment for acute large vessel occlusion stroke, endorsed by European Stroke Organisation and European Society for Minimally Invasive Neurological Therapy recommendations [4], [5]. We describe a case of periprocedural ischemic stroke during TAVI, promptly managed with mechanical thrombectomy, underscoring the importance of cardiologists' peripheral interventional competence, the role of multidisciplinary teamwork, and the evidence-based consideration of cerebral embolic protection.

## Case report

**Fig. 1:**
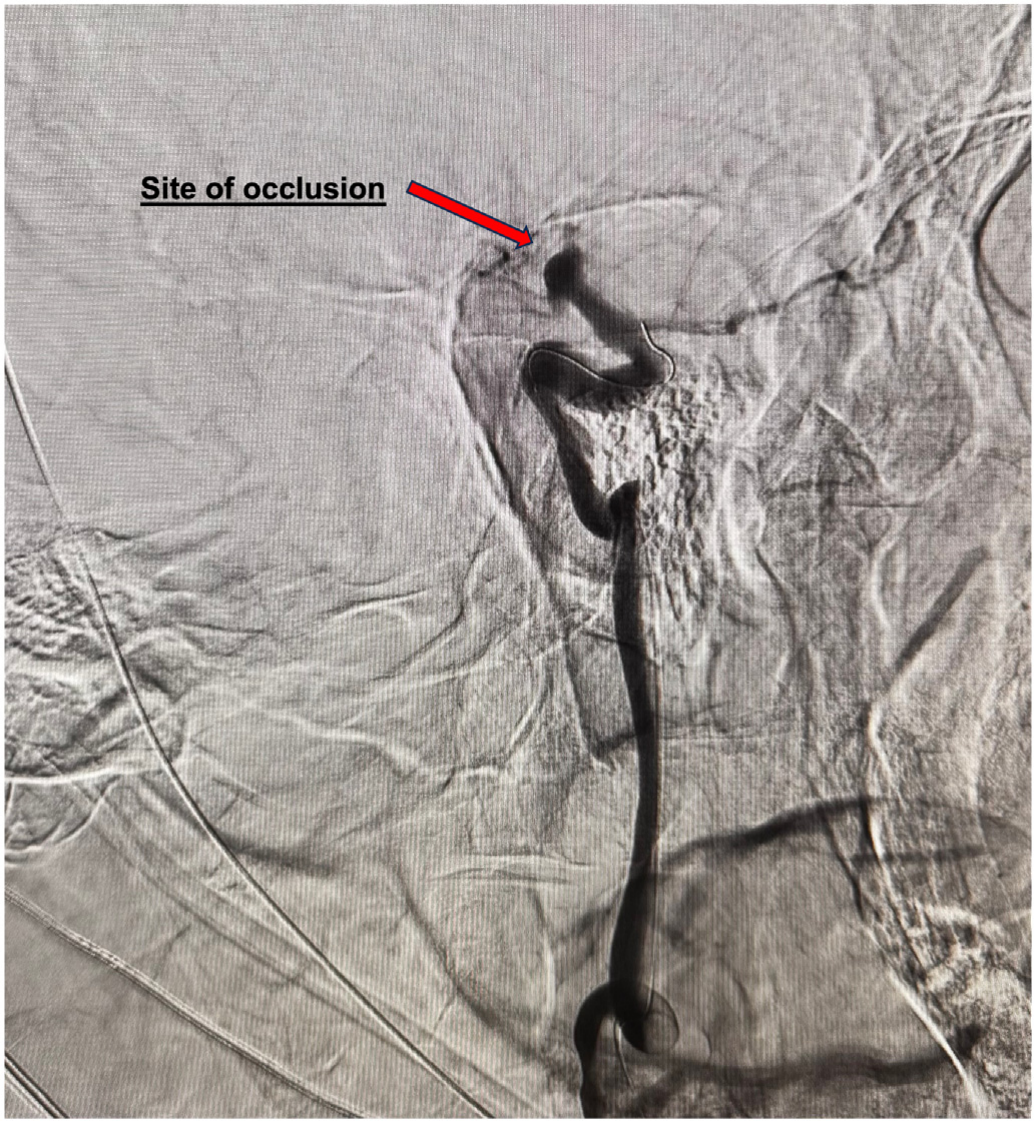
Cerebral angiography before thrombectomy showing occlusion of the right middle cerebral artery (M1 segment).

**Fig. 2:**
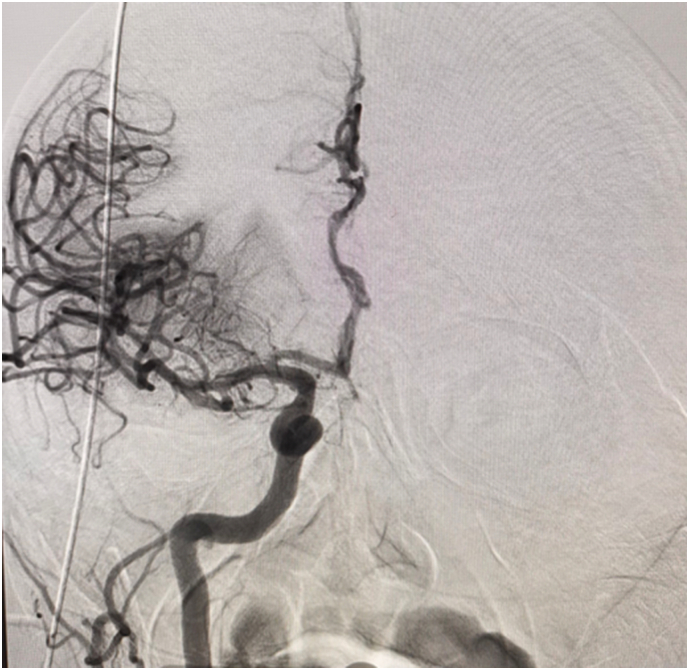
Post-thrombectomy cerebral angiography demonstrating complete recanalization (modified Thrombolysis in Cerebral Infarction grade 3).

**Fig. 3:**
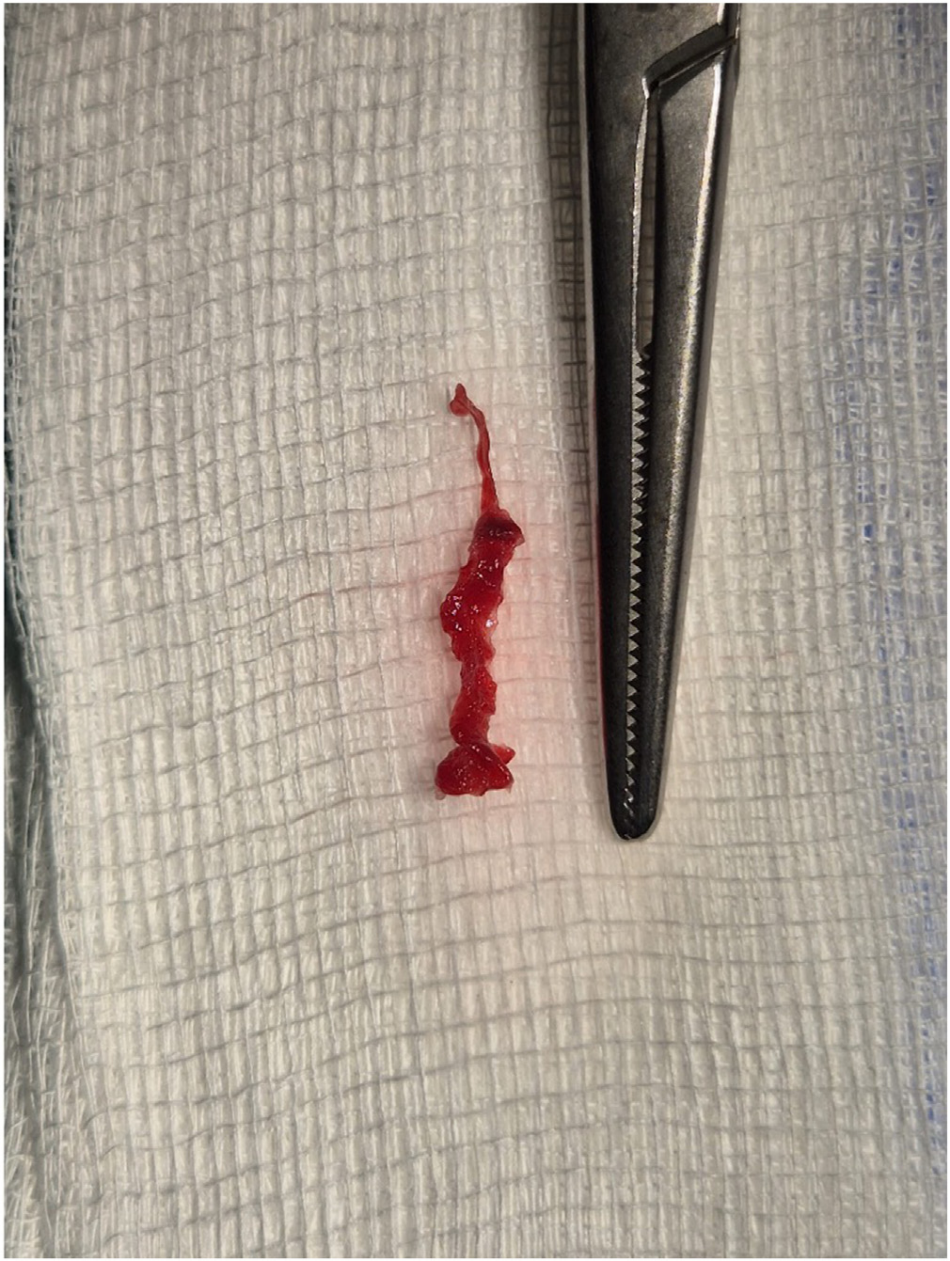
Gross appearance of the embolus retrieved during mechanical thrombectomy.

An 82-year-old female with bicuspid aortic stenosis type 1 was admitted for transfemoral TAVI with a self-expanding prosthesis (CoreValve Evolut FX+, Medtronic, Santa Rosa, CA, USA). The procedure was performed under local anesthesia with conscious sedation, allowing continuous neurological monitoring throughout the intervention. Periprocedural anticoagulation consisted of 5000 IU of intravenous unfractionated heparin administered after femoral access, targeting an activated clotting time (ACT) between 250 and 300 s according to institutional protocol. ACT was monitored during the procedure and remained within the target range. The patient was on single antiplatelet therapy with aspirin 100 mg daily and was not receiving oral anticoagulation. No heparin reversal with protamine was performed at the end of the procedure. After balloon pre-dilatation with a 22 mm VACS III balloon (OSYPKA AG, Rheinfelden, Germany), the patient suddenly developed left hemibody weakness and aphasia. The prosthetic valve was immediately implanted to secure hemodynamic stability, and subsequent aortic angiography confirmed satisfactory valve position and performance. At that point, suspecting an acute cerebrovascular event, the interventional cardiologist proceeded with carotid angiography using a JR-4 6F diagnostic catheter (Cordis, Hialeah, FL, USA), which revealed an occlusion of the M1 segment of the right middle cerebral artery. A BMW wire (Abbott Vascular, Santa Clara, CA, USA) was carefully advanced, restoring flow in the right ophthalmic artery. The stroke team was simultaneously activated, and interventional radiologists rapidly initiated mechanical thrombectomy. Using a NeuronMax 90 cm guiding catheter (Penumbra, Alameda, CA, USA), a Catalyst 6 aspiration catheter (Stryker Neurovascular, Fremont, CA, USA), and a Synchro microguidewire (Stryker Neurovascular), a single aspiration pass was performed, resulting in complete recanalization, with a modified Thrombolysis in Cerebral Infarction grade 3 within 30 min of symptom onset. The patient was stabilized, and post-procedural computed tomography excluded hemorrhagic transformation. Cerebral angiography before and after thrombectomy confirmed restoration of flow (Fig. 1, Fig. 2). The retrieved embolus was preserved and submitted to the pathology department for analysis (Fig. 3), which revealed an acute thrombotic formation, consistent with a fresh fibrin-platelet thrombus. Neurological deficits improved significantly immediately after the procedure, with near-complete resolution before hospital discharge.

## Discussion

Periprocedural stroke complicates ~2–4% of TAVI procedures [1], [2]. Despite advances in valve design and operator expertise, the incidence has remained stable over the past decade. Strokes occur most frequently in the first 48 h, often related to embolization of calcific or thrombotic debris during valve manipulation [2]. The prognostic impact is severe. Eggebrecht et al. reported a 30-day stroke/transient ischemic attack incidence of 3.3% across >10,000 patients, with mortality more than threefold higher in patients experiencing stroke [1]. Vlastra et al., in a pooled patient-level analysis of 10,982 transfemoral cases, confirmed a 2.4% 30-day stroke rate and a sixfold increase in mortality among those affected [2]. In Medicare data (>129,000 TAVI patients), stroke was associated with higher one-year mortality, recurrent events, and increased healthcare utilization [3]. Management of acute ischemic stroke complicating TAVI has been reviewed in recent systematic analyses. Hammond-Haley et al. demonstrated that mechanical thrombectomy is feasible, safe, and effective, while intravenous thrombolysis is associated with significant bleeding risk after large-bore access and may not be effective in the case of calcific or device-related emboli [6]. Cerebral embolic protection (CEP) has been proposed as a preventive measure. However, randomized evidence does not support its routine use. The PROTECT-TAVI trial, including 7635 patients, found no reduction in periprocedural stroke with routine CEP [7]. Similar results emerged from PROTECTED TAVR [8]. These data support the fact that in our case, despite the absence of cerebral protection, the outcome was favorable due to swift recognition and management. ESC guidelines therefore do not recommend systematic CEP, but rather emphasize center readiness for rapid complication management [4]. Recent evidence has emphasized that the management of acute ischemic stroke complicating TAVI remains poorly standardized, with limited data on emergency neurointervention [9]. In the large Vizient database including 1135 post-TAVI strokes, only 4.4% of patients underwent mechanical thrombectomy, yet neurointervention was associated with a threefold higher likelihood of disability-free survival compared with conservative management [10]. Bansal et al. [9] proposed a structured management algorithm highlighting the critical role of early neurological assessment under conscious sedation, prompt activation of the institutional “code stroke” pathway, and rapid brain imaging to determine candidacy for thrombectomy. Our case aligns with this approach, as the interventional cardiologist's immediate carotid angiography enabled swift identification of large-vessel occlusion and expedited multidisciplinary treatment within the optimal therapeutic window. Although mechanical thrombectomy following TAVI has been previously described, in many reported cases diagnosis follows patient transfer for neuroimaging. In our case, the ability of the structural operator to perform immediate carotid angiography within the catheterization laboratory allowed real-time identification of large-vessel occlusion, potentially reducing diagnostic delay and expediting reperfusion. This intra-laboratory diagnostic capability represents a relevant operational aspect in contemporary TAVI programs. This competence, although not universally emphasized, should be recognized as fundamental for operators performing complex structural interventions. Multidisciplinary collaboration was central to the successful outcome: interventional cardiologists, neurologists, and interventional radiologists worked seamlessly, reflecting the ESC recommendation that TAVI should only be performed in centers with access to comprehensive teams and immediate neurovascular rescue. These findings further support the integration of well-defined neurointerventional protocols and intra-procedural vigilance in contemporary TAVI programs.

Finally, histopathological examination of the retrieved embolus demonstrated an acute thrombotic formation, confirming that the cerebrovascular event was caused by fresh thrombotic material rather than calcific or tissue-derived debris. This finding supports the hypothesis that thrombotic embolization may occur during valve preparation or manipulation, particularly in the setting of balloon pre-dilatation and temporary cessation of flow. Recognition of the acute thrombotic nature of the embolus in our case underscores the importance of meticulous antithrombotic management and procedural vigilance to minimize periprocedural thromboembolism.

## Conclusion

This case demonstrates that periprocedural stroke during TAVI, although rare and potentially catastrophic, can be effectively managed if promptly recognized. Immediate carotid angiography by the interventional cardiologist, rapid activation of the stroke team, and timely mechanical thrombectomy resulted in complete recovery. The absence of cerebral embolic protection, consistent with the latest randomized evidence, did not compromise the outcome. Ensuring peripheral angiographic skills and fostering multidisciplinary collaboration remain essential to optimize safety during structural interventions.

## Consent statement

Written informed consent was obtained from the patient for publication of this case report, including accompanying images.

## Funding

This research did not receive any specific grant from funding agencies in the public, commercial or not-for-profit sectors.

## Declaration of competing interest

The authors declare no conflicts of interest related to this work.
